# Fatigue as main symptom in an elderly multiple myeloma patient: a Case Report

**DOI:** 10.3389/fmed.2025.1635403

**Published:** 2025-08-18

**Authors:** Xuanna Li, Shuang Li, Junrong Chen

**Affiliations:** ^1^Department of General Medicine Practice, The Sixth Affiliated Hospital, Sun Yat-sen University, Guangzhou, Guangdong, China; ^2^Biomedical Innovation Center, The Sixth Affiliated Hospital, Sun Yat-sen University, Guangzhou, China

**Keywords:** fatigue, general practice, multiple myeloma, extramedullary, case report

## Abstract

**Introduction:**

The high prevalence of fatigue as an atypical clinical manifestation in general practice creates notable diagnostic challenges, particularly in geriatric patients.

**Methods:**

We describe a case of an 80-year-old female presenting with fatigue, initially attributed to poorly controlled diabetes mellitus or upper respiratory infection, who was ultimately diagnosed with multiple myeloma after admitted to the Department of General Medicine at a general hospital. Initial diagnostic workup including physical examination revealed the presence of anterior thoracic mass with undetermined etiology. Laboratory analysis demonstrated characteristic hematological abnormalities: normocytic anemia (hemoglobin 91 g/L), significant hyperglobulinemia (serum globulin 76.18 g/L), and hypoalbuminemia (serum albumin 27.94 g/L), showing a reversed albumin-globulin (A/G) ratio of 0.37. Guided by Murtagh’s safe diagnostic strategy, comprehensive imaging studies including contrast-enhanced cervical, thoracoabdominal computed tomography (CT) and 18F-FDG PET-CT were performed. Histopathological confirmation was obtained from the gastric mass (CD138^+^/CD38^+^ plasma cell infiltration) and anterior mediastinal tumor (*κ* light chain restriction). Serum immunofixation electrophoresis revealed monoclonal IgA-*κ* paraprotein, while bone marrow aspiration demonstrated 80% clonal plasma cells. These findings collectively fulfilled the International Myeloma Working Group (IMWG) diagnostic criteria for multiple myeloma with extramedullary involvement.

**Results:**

The patient was transferred to Hematology Department for further therapy. Clinical reassessment at 6-week follow-up showed symptomatic improvement and anterior thoracic mass regression.

**Discussion:**

We report a diagnostic case study of a MM patient whose initial presentation was fatigue, and clinicians should prioritize the overall condition, reduce diagnostic delays and improve therapeutic outcomes through early intervention strategies.

## Introduction

Fatigue, as one of the most common symptoms of unexplained symptoms, is a frequent chief complaint in general practice clinics of comprehensive hospitals. According to a study by MAISEL P et al., fatigue accounts for approximately 10–20% of all consultations with a primary care physician (1). As an atypical symptom, especially in elderly population, fatigue may stem from either physiological or pathological causes. Physiological fatigue primarily refers to self-perceived exhaustion resulting from aging, work overload, stress, sleep deprivation, and similar factors, which can typically be alleviated or resolved with adequate rest and emotional adjustment. In contrast, pathological fatigue may be associated with various systemic disorders and requires careful clinical differentiation due to its diverse etiologies (2). Fatigue often poses diagnostic challenges in the clinical setting, particularly cancer-related fatigue, which is frequently underrecognized and leads to delays in both diagnosis and treatment.

Multiple myeloma (MM), as the second most prevalent hematologic malignancy, exhibits an incidence rate of 1.60 per 100,000 population (3–5). MM typically presents with an insidious onset and lacks disease-specific symptoms, such as fatigue, in its early stages, often leading to misdiagnosis or delayed diagnosis in clinical practice. Early detection and intervention are crucial for improving prognosis, enhancing quality of life, and prolonging survival (6). The promulgation of evidence-based consensus guidelines has driven heightened clinical recognition of MM (7, 8). Although international diagnostic and treatment guidelines have enhanced specialists’ understanding of MM, general practitioners (GPs) often exhibit limited awareness and low clinical suspicion, contributing to diagnostic delays.

This article reports a diagnostic case study of a MM patient whose main complaint was fatigue, aiming to: (1) Optimize general practitioners’ diagnostic detection of multiple myeloma and associated extramedullary disease; (2) illustrate the utility of Murtagh’s safe diagnostic strategy in evaluating atypical symptoms; and (3) emphasize the significance of health management models in facilitating accurate diagnosis and treatment.

## Case presentation

**Figure 1:**
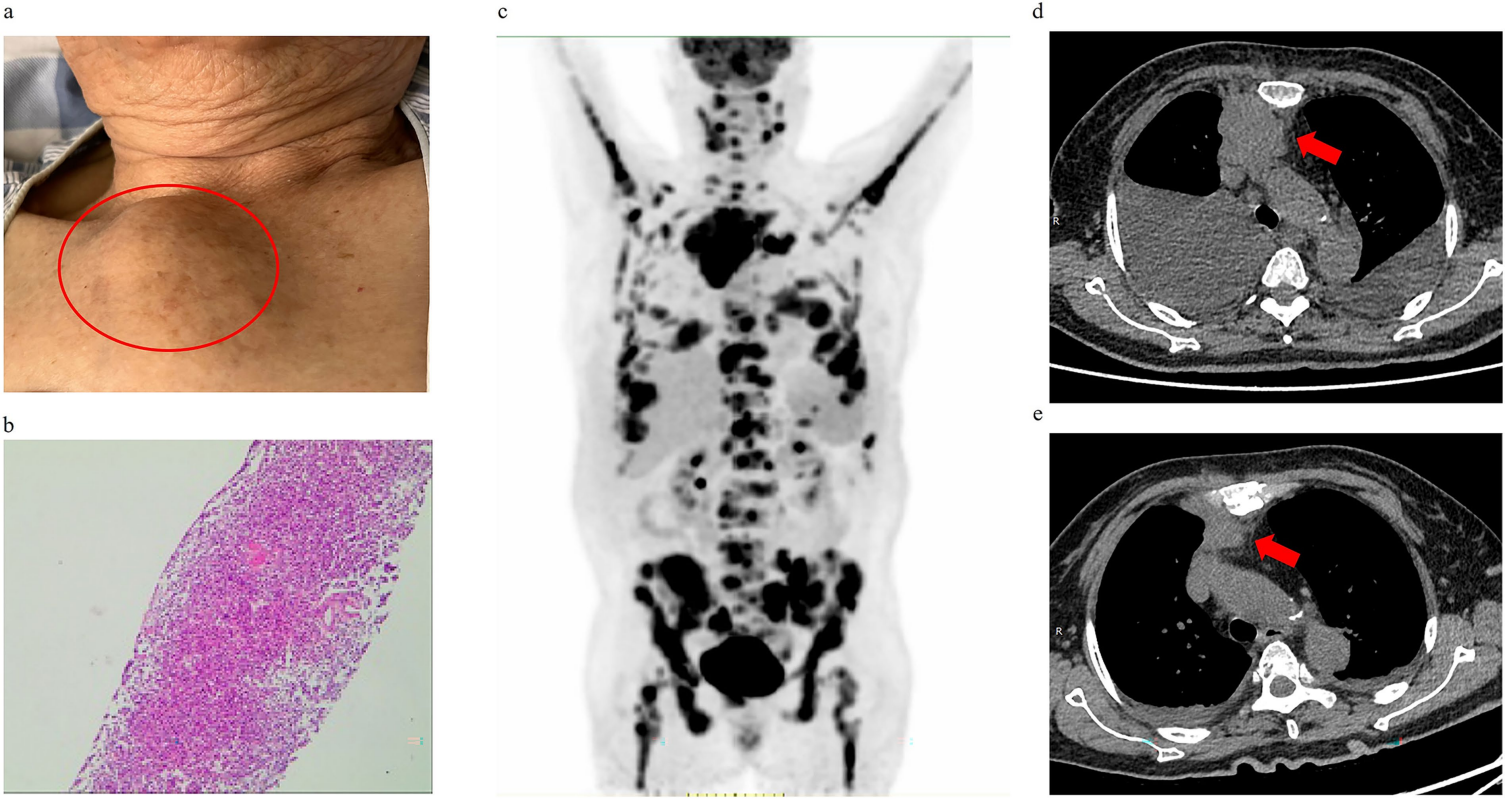
Diagnostic confirmation of extramedullary involvement in multiple myeloma via anterior thoracic mass evaluation. **(a)** Physical examination on admission revealed a palpable anterior thoracic mass. **(b)** Hematoxylin–eosin (H&E) staining of anterior thoracic mass biopsy demonstrated diffuse sheet-like proliferation of neoplastic cells. **(c)** 18F-FDG PET-CT imaging (6.37 mCi dose) showing hypermetabolic foci in anterior mediastinum, stomach, pancreas, and bilateral pleura. **(d)** Pre-therapeutic anterior mediastinal mass (red arrow). **(e)** Post-treatment anterior mediastinal mass (red arrow).

**Figure 2:**
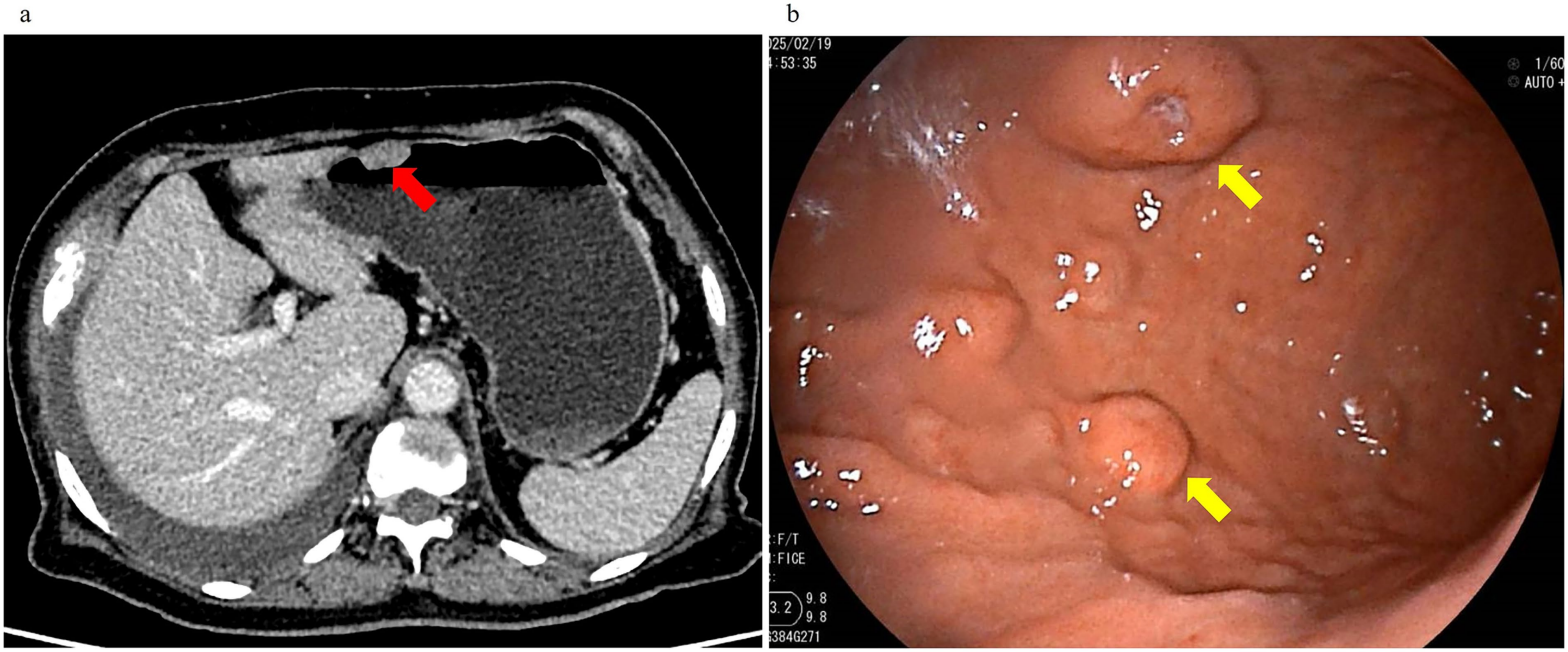
Enhanced abdominal CT and gastroscopy revealed gastric masses. **(a)** Contrast-enhanced CT demonstrated a submucosal irregular nodular protrusion with homogeneous enhancement (red arrow). **(b)** Gastroscopy showed scattered elevated masses (×40 magnification, yellow arrow).

**Figure 3:**
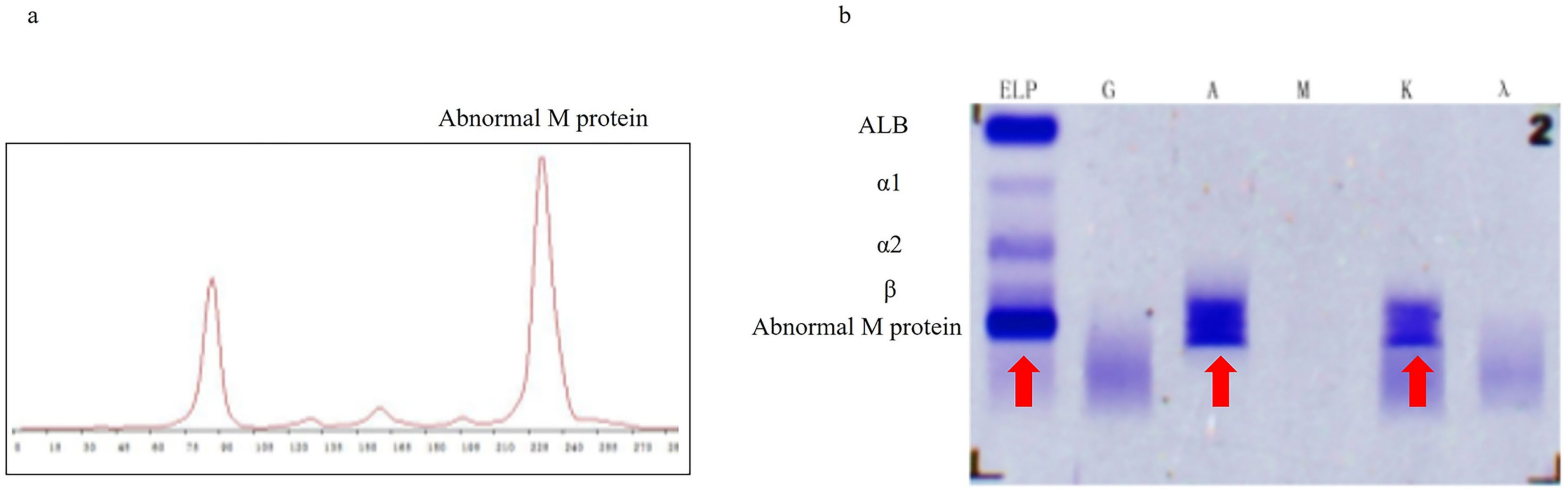
Serum tests revealed the following characteristics: **(a)** Serum protein electrophoresis demonstrated a suspected abnormal M-protein in the β2 region. **(b)** Serum immunofixation electrophoresis showed dense bands (red arrows) in the ELP lane, as well as the A (IgA) and K (*κ*) regions.

**Figure 4:**
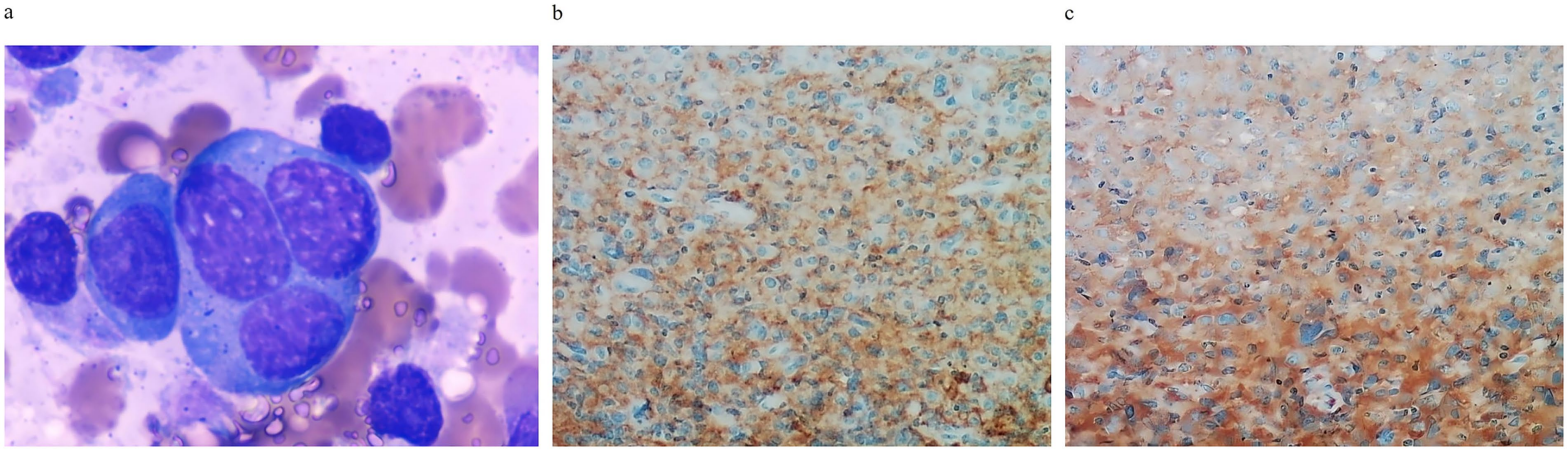
Histopathological findings of bone marrow at diagnosis. **(a)** Plasma cell morphology showing atypical plasma cells (×1,000 magnification). **(b)** CD38 immunohistochemistry demonstrating plasma cell infiltration (×20 magnification). **(c)** Kappa light chain restriction with positive plasma cell infiltration (×20 magnification).

An 80-year-old woman with a history of hypertension and diabetes mellitus presented with a one-month history of progressive fatigue, accompanied by an episode of hypoglycemic coma (fingerstick glucose: 2.2 mmol/L) and productive cough. Prior evaluation at other hospitals attributed fatigue symptom to suboptimal glycemic control and upper respiratory tract infection (URTI) without thorough physical examination and initial blood tests, yet her condition failed to improve with blood glucose level and URTI symptoms controlled. Additionally, the patient reported 7 kg of unintentional weight loss over the preceding year, which she attributed to strict dietary control for diabetes mellitus (DM). Following an initial evaluation at our general medicine clinic for persistent unexplained fatigue, the patient was arranged hospitalization to expedite comprehensive etiological investigations. Upon admission, comprehensive health problem and risk assessment was performed (Table 1). Physical examination during hospitalization revealed a previously undetected 10 × 10 cm fixed, firm anterior thoracic mass (Figure 1a) and subtle signs of anemia. Laboratory investigations demonstrated clinically significant abnormalities: normocytic anemia (hemoglobin 91.0 g/L), pronounced hyperglobulinemia (globulin 76.18 g/L) with concomitant hypoalbuminemia (albumin 27.94 g/L), and elevated tumor markers (CA-125: 98.60 U/mL; CA 19–9: 38.87 U/mL). Guided by Murtagh’s safe diagnostic strategy, the constellation of advanced age, anemia, and a thoracic mass was highly indicative of an underlying malignancy. Given the anterior thoracic mass, contrast-enhanced CT scans of the neck, chest, and abdomen were performed. These revealed findings suggestive of an anterior mediastinal malignant tumor (Figure 1d), multiple gastric protrusions (Figure 2a), and further gastroscopic examination revealed multiple protruding gastric masses (Figure 2b). Elevated tumor markers further supported the likelihood of a malignant anterior mediastinal process. The diagnostic evaluation prioritized differentiating between a solid tumor and hematological malignancy as the etiology of the anterior mediastinal mass. Concurrently, atypical infections (e.g., tuberculosis, fungal) were excluded based on negative results of the following assays: Purified Protein Derivative (PPD) skin test, T-SPOT. TB (T-cell spot forming assay), serum (1,3)-*β*-D-glucan, and serum galactomannan, combined with normal levels of C-reactive protein and procalcitonin. Further diagnostic workup included serum protein electrophoresis, serum immunofixation electrophoresis, 18F-FDG PET-CT (Figure 1c), gastroscopy with gastric biopsy, ultrasound-guided core needle biopsy of the anterior thoracic mass, and bone marrow histopathological examination. Serum analysis revealed a significant elevation of immunoglobulin A (IgA) to 39.56 g/L, serum protein electrophoresis detected a suspected M-protein band in the β2 region (Figure 3a). Immunofixation confirmed an IgA-*κ* monoclonal immunoglobulin (Figure 3b). Bone marrow biopsy demonstrated findings consistent with plasma cell myeloma, with CD38-positive myeloma cells comprising 80% of clonal plasma cells and κ light chain restriction (Figure 4). Pathological examination confirmed plasmacytoma in both gastric and anterior mediastinal masses (Figure 1b). The patient was diagnosed with IgA-κ multiple myeloma with extramedullary involvement (mediastinum and gastrointestinal tract). She was transferred to the Department of Hematology and received first-line therapy with the DRd regimen (daratumumab, lenalidomide, and dexamethasone). At the 6-week clinical follow-up, fatigue demonstrated improvement, the mediastinal mass exhibited a reduction in size (Figures 1d,e), serum IgA decreased from 39.56 g/L to 0.5 g/L, and serum M-protein (IgA-*κ*) decreased from 16.77 g/L to 3.36 g/L. The patient reported satisfaction with her clinical status and continued regular treatment and follow-up.

**Table 1:** Health problems and risk assessment details.

**Current health problems**	**Findings**
Diabetes mellitus	Pancreatic islet function: Not assessed; Diabetic complications: Not evaluated; Glycemic control: Poor (elevated fasting glucose + hypoglycemic coma episodes)
Hypertension	Hypertensive complications: Not assessed; Antihypertensive therapy: Levamlodipine besylate; BP control: Maintained (130–140/70–80 mmHg)
Respiratory symptoms	Respiratory symptoms: Productive cough; Clinical evaluation: Not performed
anterior thoracic mass	Clinical detection: Negative (patient-reported, family-observed, and clinician-assessed)
Fatigue	Clinical progression: Worsening; Family awareness: No; Fatigue cause: Unidentified

**Demographics**	**Findings**
Age	80 years (geriatric)

**Clinical assessments**	**Findings**
Nutritional risk screening	Score: 1
Fall risk	High risk
VTE risk	Score: 1, low risk
Bleeding risk	Low risk

**Lifestyle factors**	**Findings**
Physical activity	Daily walking
Diet	Balanced diet with controlled carbohydrate intake

**Psychological status**	**Findings**
Mental health	Avoidance of health discussion to minimize family distress
Treatment adherence	Good compliance with limited disease awareness

**Social context**	**Findings**
Occupation	Retired
Family support	Widowed, cohabiting with children
Financial status	Covered by medical insurance
Social support	Adequate community support

## Discussion

### Murtagh’s safe diagnostic strategy guides the etiological investigation of fatigue

In 2003, John Murtagh proposed a diagnostic framework for fatigue, known as the Murtagh’s safe diagnostic strategy. This approach enables clinicians to systematically evaluate potential causes ranging from life-threatening conditions to rare diseases and systemic disorders, thereby improving diagnostic accuracy and reducing the risk of missed diagnoses in clinical practice (9, 10).

This elderly female patient with multiple comorbidities (diabetes mellitus, hypertension, respiratory infection, and anterior thoracic mass) underwent comprehensive health problem and risk assessment upon admission (Table 1). Presenting with fatigue as the chief complaint, pathological fatigue was considered highly probable based on differential diagnosis and risk evaluation (2). Murtagh’s safety diagnostic strategy was applied upon hospital admission (Table 2).

**Table 2:** Application of Murtagh’s safe diagnostic strategy.

**Probable diagnoses:** The patient exhibited chronic non-remitting fatigue with poorly controlled diabetes and hypertension, accompanied by respiratory infection, weight loss. The complex presentation involving endocrine, cardiovascular, and respiratory systems warranted hospitalization for further investigation.
**Red flag conditions:** Initial evaluation ruled out acute cardiovascular events (normal cardiac workup) and severe infection (negative CRP/PCT). However, physical examination revealed anterior thoracic mass, laboratory findings revealed mild anemia (Hb 91 g/L), hyperglobulinemia (Glb 76.18 g/L), hypoalbuminemia (Alb 27.94 g/L), and elevated ESR (137.68 mm/h). These abnormalities raised concern for occult malignancy, particularly cancer-related fatigue (CRF).
**Commonly overlooked conditions:** Given the patient’s vascular risk factors (advanced age, diabetes, hypertension) and recent respiratory infection, cerebrovascular disease, chronic infections, and medication side effects required consideration.
**Masked pathologies:** Atypical infections (e.g., tuberculosis) were included in the differential diagnosis.
**Psychosocial factors:** The patient’s recent widowhood and tendency to minimize symptoms (“concealing distress while reporting wellness”) necessitated evaluation for undiagnosed depression or unexpressed somatic complaints. Close monitoring of subjective experiences was imperative.

Under the guidance of Murtagh’s safe diagnostic strategy, we systematically completed the diagnostic workup, ultimately establishing the diagnosis of IgA-*κ* type multiple myeloma with multiorgan extramedullary involvement (mediastinum and gastrointestinal tract). This case demonstrates that GPs can effectively utilize Murtagh’s safety diagnostic strategy in clinical differential diagnosis to reduce rates of delayed or missed diagnoses.

### Multiple myeloma presenting with fatigue and rare extramedullary disease carries high risk of missed diagnosis

Multiple myeloma (MM) presenting primarily with fatigue is prone to missed diagnosis in clinical practice, particularly when accompanied by extramedullary disease (EMD) at rare sites such as the mediastinum or gastrointestinal tract. MM is a hematologic malignancy involving plasma cells, primarily characterized by clonal proliferation of abnormal plasma cells leading to organ dysfunction. The typical clinical manifestations, known as “CRAB” symptoms, include: calcium elevation, renal insufficiency, anemia, bone disease, as well as secondary amyloidosis and related complications (11). MM may also give rise to clones that grow independent of the bone marrow niche and invade other organs, known as extramedullary disease (EMD). The reported incidence of EMD in newly diagnosed MM ranges from 0.5 to 4.8%, with preferential involvement of skin and soft tissues. In contrast, gastric and mediastinal infiltration exhibits significantly lower frequency (12). Statistical data for rare EMD sites such as mediastinum are currently lacking in the literature. The development of EMD in MM patients significantly impacts both prognosis and therapeutic outcomes, underscoring the critical importance of early detection and diagnosis. Diverse clinical manifestations, limited awareness of disease-related manifestations, and suboptimal diagnostic strategies often lead to diagnostic delays in MM, especially in GPs (12–14).

This case underscores the diagnostic challenges in elderly patients presenting with fatigue as the predominant symptom, wherein the condition remained unrecognized for over a month across multiple clinical visits for various ailments including respiratory infections, hypoglycemic episodes, and poorly managed diabetes, owing to attribution to age-related comorbidities, patient underestimation of symptoms, and clinical oversight. Notably, this study characterizes rare occurrences of EMD at non-classical sites, such as mediastinum and gastrointestinal tract, in multiple myeloma, expanding the phenotypic landscape and underscoring diagnostic vigilance for atypical manifestations. Furthermore, this case identifies two diagnostic imperatives: optimizing geriatric health literacy through patient education, and synthesizing clinical findings from physical assessments and core diagnostics to prevent treatment-delaying misdiagnoses.

### General practitioners’ clinical reasoning provide diagnostic value for identifying hidden hematologic malignancies

As health gatekeepers, GPs assess health problems and evaluate risks to manage community-dwelling patients with prevalent chronic conditions (15). In clinical practice, GPs may similarly employ health management strategies to aid in diagnosis. In this case, the GPs’ systematic assessment during diagnose process revealed three clinically significant elements: (1) palpable anterior thoracic mass, (2) elderly individuals deliberately concealing their medical conditions, and (3) progressive fatigue. These findings provided crucial clues for final diagnosis.

In general practice, patients commonly present with non-specific symptoms including fatigue and unintentional weight loss, creating substantial diagnostic dilemmas (16). For GPs, refining systematic diagnostic approaches while improving recognition of rare diseases is essential to facilitate timely diagnosis, prevent diagnostic errors, and mitigate management delays (17). In this case, accurate diagnosis was achieved promptly through comprehensive health assessment, systematic physical examination, and implementation of Murtagh’s safe diagnostic strategy, enabling appropriate therapeutic interventions.

## Conclusion

In summary, this elderly patient presenting with non-specific fatigue underwent multiple evaluations in community clinics and emergency departments without obtaining a definitive diagnosis, which was ultimately established by general practitioners at a general hospital. This diagnostic trajectory underscores both the challenges and critical importance of timely diagnosis based on non-specific symptoms in general practice. As a prevalent hematologic malignancy, multiple myeloma frequently manifests with protean systemic manifestations requiring holistic clinical assessment. This case highlights the imperative for enhanced clinical reasoning training to facilitate early diagnosis, prompt treatment initiation, and optimized patient outcomes.

## Data Availability

The original contributions presented in the study are included in the article/supplementary material, further inquiries can be directed to the corresponding author/s.
